# Using floating catchment area (FCA) metrics to predict health care utilization patterns

**DOI:** 10.1186/s12913-019-3969-5

**Published:** 2019-03-04

**Authors:** Paul L. Delamater, Ashton M. Shortridge, Rachel C. Kilcoyne

**Affiliations:** 1 Department of Geography and the Carolina Population Center, University of North Carolina at Chapel Hill, Chapel Hill, NC 27599 USA; 2 Department of Geography, Environment, and Spatial Sciences, Michigan State University, East Lansing, MI 48824 USA; 3 Department of Geography and Geoinformation Science, George Mason University, Fairfax, VA 22030 USA

**Keywords:** Spatial accessibility, Access to health care, Health care use, Utilization patterns, Hospitalizations, Floating catchment areas

## Abstract

**Background:**

Floating Catchment Area (FCA) metrics provide a comprehensive measure of potential spatial accessibility to health care services and are often used to identify geographic disparities in health care access. An unexplored aspect of FCA metrics is whether they can be useful in predicting where people actually seek care. This research addresses this question by examining the utility of FCA metrics for predicting patient utilization patterns, the flows of patients from their residences to facilities.

**Methods:**

Using more than one million inpatient hospital visits in Michigan, we calculated expected utilization patterns from Zip Codes to hospitals using four FCA metrics and two traditional metrics (simple distance and a Huff model) and compared them to observed utilization patterns. Because all of the accessibility metrics rely on the specification of a distance decay function and its associated parameters, we conducted a sensitivity analysis to evaluate their effects on prediction accuracy.

**Results:**

We found that the Three Step FCA (3SFCA) and Modified Two Step FCA (M2SFCA) were the most effective metrics for predicting utilization patterns, correctly predicting the destination hospital for nearly 74% of hospital visits in Michigan. These two metrics were also the least sensitive to changes to the distance decay functions and parameter settings.

**Conclusions:**

Overall, this research demonstrates that FCA metrics can provide reasonable predictions of patient utilization patterns and FCA utilization models could be considered as a substitute when utilization pattern data are unavailable.

**Electronic supplementary material:**

The online version of this article (10.1186/s12913-019-3969-5) contains supplementary material, which is available to authorized users.

## Background

Much of the recent geographic research regarding access to health care has focused on examinations of *potential* access to services, rather than on *realized* access or utilization of health care services [1]. As defined by Aday and Anderson [2], potential access may be considered as a measure of the potential for entry into the health care system or a characterization of the level of opportunity provided by the health care delivery system. Conversely, realized access is a measure of actual utilization of a health care service, such that any barriers to the use of services have been overcome and access has been achieved. Penchansky and Thomas [3] further examined the concept of potential access, providing five distinct dimensions of access, which Khan [4] later categorized into spatial components (accessibility and availability) and aspatial components (affordability, acceptability, and accommodation).

The fusion of accessibility (distance to services) and availability (volume of services) has been termed *spatial accessibility* [5]. The Floating Catchment Area (FCA) family of metrics simultaneously integrate the three essential components required to measure potential spatial accessibility: supply of services, potential demand for services, and distance separating supply and demand locations. Much of the recent FCA-related research has focused on methodological improvements to the metrics (e.g., [6–8]) or using the metrics to map and identify disparities in health care accessibility (e.g., [9–11]).

Less effort has been dedicated to understanding how potential spatial accessibility affects health care utilization. While there certainly are exceptions to this statement (e.g., [12–15]), the emphasis of potential spatial accessibility research has largely remained on “potential”. Ngui and Apparcio [16] note the complex nature of incorporating potential and realized information within a single study design, which may be one factor limiting research in this area. However, this deficit could simply be a result of differences in data availability; detailed health care utilization data is not always readily available to researchers due to privacy concerns, while health care facility locations and geographic population data are relatively easy to obtain.

People who live in the same region may utilize care at numerous facilities. Data representing the number of people residing in each region who use health care at multiple facilities are referred to as *utilization patterns* or *patient flows*. For a study area partitioned into *n* regions based on people’s residence and having *m* facilities located within it, utilization patterns are expressed as an *n* x *m* Origin-Destination (OD) matrix with the matrix entries containing the number of visits or volume of use for residents of each region at each facility. Previous research on health care utilization patterns has generally been explanatory in nature, focusing on identifying whether population and facility characteristics, as well as distance, affect where people receive care (e.g., [17–23]); less emphasis has been placed on developing models that provide accurate predictions of patient utilization patterns.

In this research, we evaluated the potential utility of using measures of spatial accessibility, namely FCA metrics, to predict spatial patterns of health care utilization. Interestingly, the FCA metrics contain the requisite information to predict utilization patterns; however, they have yet to be evaluated in this capacity. We tested four FCA metrics, the Enhanced Two Step FCA (E2SFCA, [24]), the Modified 2SFCA (M2SFCA, [25]), the Three Step FCA (3SFCA, [26]), and a Huff-modified version of the 3SFCA (abbreviated H3SFCA for this work, [27]). Two traditional spatial accessibility approaches are implemented for comparative purposes: a simple distance-based approach and a Huff Model [28]. Each measure is highly dependent on the definition of a distance decay function and its parameter values. Thus, for each metric, we implemented four different distance decay functions, each having four parameter settings, to test for how characterization of distance decay affects the predictive accuracy of the metrics. Our approach produced a total of 96 outputs (6 metrics × 4 decay functions × 4 parameter settings) that were compared against observed utilization patterns. We also examined one single metric, decay function, and parameter setting combination in detail to demonstrate the general nature of where the predictions were most and least accurate to better understand the spatial distribution of predictive accuracy and factors that may have influenced it.

## Methods

### Input data and preprocessing

Our case study was conducted using inpatient hospitalizations and hospitals in the state of Michigan (US). We evaluate all general acute care hospitalizations over an entire year in the state, as this is a nonspecialized, relatively common type of care. Michigan makes an ideal case study for several reasons, 1) as the largest state (by area) in the eastern United States, its nearly 10 million residents live in a wide range of representative communities, from large urban cores and suburbs to rural and wilderness areas, 2) the geographic distribution of acute-care hospitals is spatially heterogeneous, leading to large variations in potential spatial accessibility, and 3) much of the state’s borders, along the Great Lakes and Canada, are effectively impassable for hospital service users, lessening the effects of study boundaries on models developed there.

Location and attribute data for hospitals in Michigan were acquired from the Michigan Department of Health and Human Services (MDHHS). The hospital attribute information was used to subset the data to only acute care hospitals offering emergency room services (*n* = 133) in an effort to remove hospitals providing only highly-specialized services that would be expected to draw patients under their own unique circumstances. The hospitalization utilization data was drawn from the 2014 Michigan Inpatient Database (MIDB), a hospital discharge database that contains, among other attributes, the residential location of the patient (at the Zip Code level) and hospital visited for each inpatient hospitalization in the state (including Michigan residents visiting Michigan hospitals, Michigan residents who visited out of state hospitals, and out-of-state residents who visited Michigan hospitals). Zip Codes were used as the spatial population unit, as this is the most resolved location information in the MIDB. The patient discharges were subset to include only in-state residents visiting in-state hospitals, heretofore referred to as in-state visits (*n* = 1,063,721). Three hospitals did not report utilization data, thus were removed from the hospital data layer and analysis. One Zip Code had no in-state visits, thus was removed from the analysis.

A roads database was acquired from the state of Michigan Open Data Portal (http://gis-michigan.opendata.arcgis.com) and subsequently converted to a vehicular travel network as described in Delamater et al. [29]. A Zip Code polygon layer was downloaded from ESRI (https://www.arcgis.com/home/item.html?id=8d2012a2016e484dafaac0451f9aea24) and subset to Zip Codes in Michigan. A small number of manual adjustments were required to ensure the Zip Code layer matched the utilization data. US Census block polygons were downloaded from the Michigan Open Data Portal and converted to a point layer, with each point representing the geographic centroid of its corresponding Census block polygon. The total population in 2010 for each block was downloaded from the US Census (https://www2.census.gov) and joined to the spatial point layer. The block population points were then spatially joined to the Zip Code polygons and used to create the population-weighted centroid (PWC) each Zip Code, as well as to calculate the total population of each Zip Code.

Using the travel network, we constructed an OD matrix containing the estimated travel time from all Zip Code PWCs to all the acute care hospitals in Michigan. The travel time data were used to subset the hospitalization data to include only visits to hospitals that were less than or equal to 90 min from the patients’ residential addresses. This step was required to remove visits that most likely occurred while the patients were away from their residence or visits that required a type of health care service that was not available in their local region. The visits data were aggregated (summed) by Zip Code and hospital and stored in an OD matrix with the Zip Codes as origins, the hospitals as destinations, and the number of visits (counts) as the entries. One Zip Code was removed from the analysis at this stage because its residents had no visits to a hospital within 90 min of the Zip Code. The final observed utilization patterns OD matrix contained 1,034,492 inpatient hospital visits (97.3% of all in-state visits), occurring at 130 hospitals and originating from 907 Zip Codes. Of the 25,795 potential unique OD pairs meeting the 90-min travel time threshold, 13,242 had at least one patient visit.

Using the visits OD matrix, we calculated the Relevance Index (*RI*) values, which normalizes for differences in the total number of visits among Zip Codes [30]. The *RI* is calculated by dividing the number of visits by residents of the Zip Code *i* to each hospital *j* (*V*_*i,j*_) by the total number of visits to all hospitals for that Zip Code (*V*_*i*_):

1 $$ {RI}_{i,j}=\frac{V_{i,j}}{V_i} $$

**Fig. 1:**
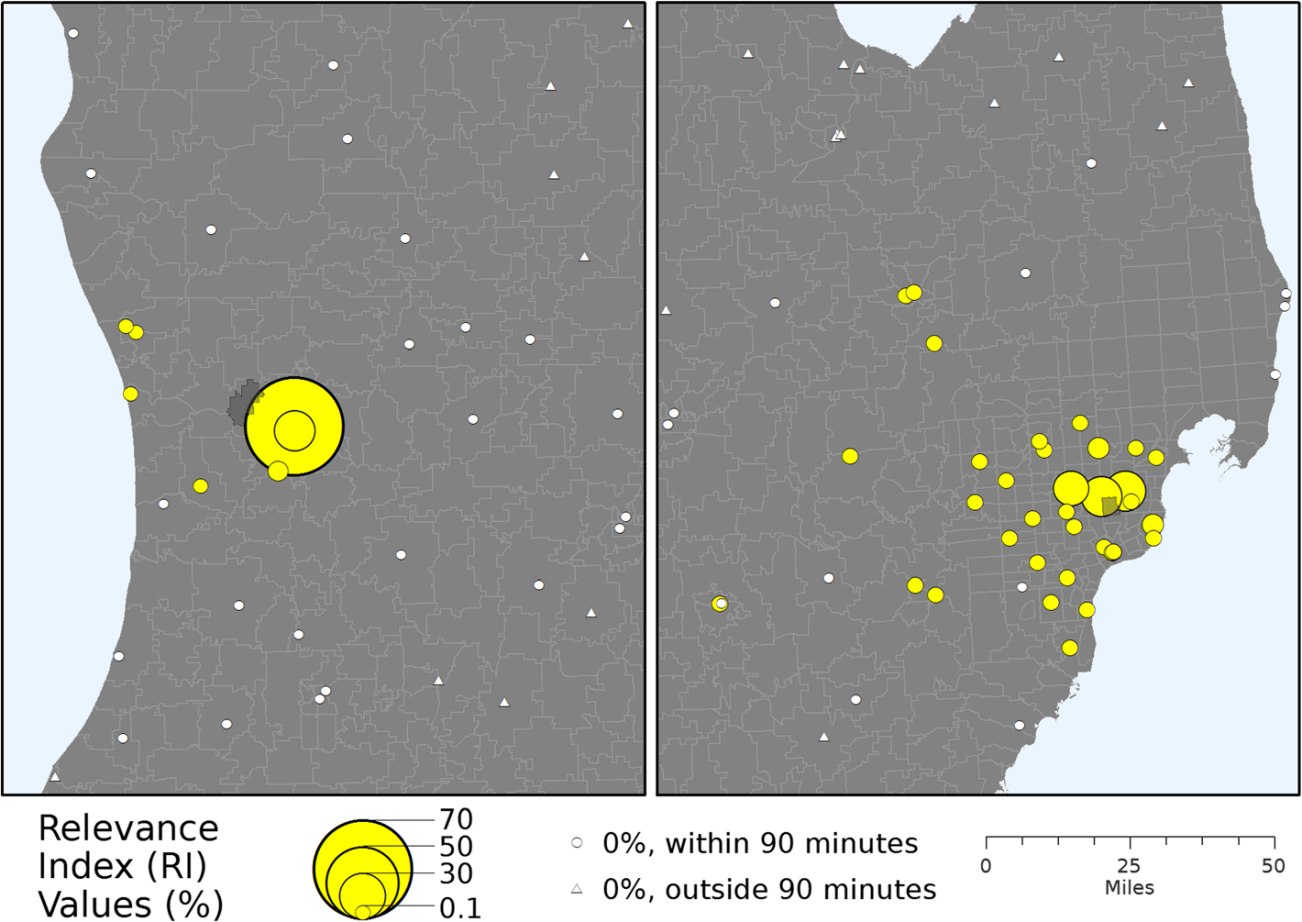
Relevance Index values (%) for two example Zip Codes

This calculation produces a set of proportion values (scaled from 0 to 1) that sums to one for each Zip Code and represents normalized utilization patterns. The *RI* values for two example Zip Codes are mapped using a population perspective in Fig. 1 to illustrate this concept, showing one Zip Code with a large proportion of visits occurring at a single facility and another Zip Code with visits more evenly dispersed across multiple facilities.

### FCA metrics

The basic framework for all floating catchment area metrics is based on a gravity model that integrates supply, demand, and distance simultaneously [31, 32]. The history of these metrics and their formulation has been extensively published in previous work (e.g., [25]) thus is only briefly summarized here. While a number of FCA metrics could have been evaluated, the following summary is limited to those used in this analysis, which were chosen because they require a similar set of data to calculate: the location of potential demand (population counts), the location of facilities and their supply, and measures of the distance separating supply and demand locations, and have similar underlying assumptions in their formulation: a single travel mode, invariant distance thresholds or catchment sizes, and total population as potential demand.

The first step in the E2SFCA is to calculate the supply to demand ratio for each facility *j* (*D*_*j*_) by dividing the supply (*S*_*j*_) by the potential demand (*P*_*j*_):

2 $$ {D}_j=\frac{S_j}{\sum_{i\in \left[{d}_{i,j}<d\right]}{P}_i{W}_{i,j}} $$

In this calculation, *P*_*j*_ is the distance-weighted sum of the population falling within a specified threshold distance (*d*) of facility *j*, *P*_*i*_ is the population at unit *i*, and *W*_*i,j*_ is the weight assigned to distance *d*_*i,j*_ based on a specified distance decay function. Common distance decay functions and their representation as weights can be found in Kwan [33] and Delamater [25], while the particular threshold distance parameter is often chosen based on the population distribution within the study region (higher threshold distances include more remote populations). The second step in the E2SFCA is to calculate the distance-weighted sum of the supply to demand ratios falling within the threshold distance of each population unit *i*:

3 $$ {A}_i={\sum}_{j\in \left[{d}_{i,j}<d\right]}{D}_j{W}_{i,j} $$

where *A*_*i*_ is the E2SFCA value.

The M2SFCA builds on the E2SFCA, but integrates an additional weight term in the formulation to account for the suboptimal distribution of supply locations. The first step is to calculate the supply to demand ratios for each facility and population unit combination (*D*_*i,j*_):

4 $$ {D}_{i,j}=\frac{S_j{W}_{i,j}}{\sum_{i\in \left[{d}_{i,j}<d\right]}{P}_i{W}_{i,j}} $$

where the rest of the terms are defined exactly as in the ESFCA. The second step in the M2SFCA is also the same as the E2SFCA, but the single facility supply to demand ratio is replaced by facility/unit value (*D*_*i,j*_):

5 $$ {A}_i={\sum}_{j\in \left[{d}_{i,j}<d\right]}{D}_{i,j}{W}_{i,j} $$

The 3SFCA and H3SFCA both attempt to account for competition among facilities by adding an additional step to the E2SFCA formulation. The first step in each metric is to first calculate a selection weight (*G*), which defines the probability that a particular facility will be selected for use by a population. In the 3SFCA, *G*_*i,j*_ for a population unit *i* and facility *j* pairing is defined as:

6 $$ {G}_{i,j}=\frac{W_{i,j}}{\sum_{j\in \left[{d}_{i,j}<d\right]}{W}_j} $$

The *G* term is simply based on distance (expressed as *W*) in the 3SFCA. The H3SFCA integrates a Huff Model to calculate *G* by incorporating both distance and supply in the formulation:

7 $$ {G}_{i,j}=\frac{S_j{W}_{i,j}}{\sum_{j\in \left[{d}_{i,j}<d\right]}{S}_j{W}_j} $$

The second and third steps of the 3SFCA and H3SFCA are the same as the two steps in the E2SFCA with the addition of the *G* term:

8 $$ {D}_j=\frac{S_j}{\sum_{i\in \left[{d}_{i,j}<d\right]}{P}_i{W}_{i,j}{G}_{i,j}} $$

and

9 $$ {A}_i={\sum}_{j\in \left[{d}_{i,j}<d\right]}{D}_j{W}_{i,j}{G}_{i,j} $$

An interesting facet of all FCA-based metrics is that the final step includes, for each population unit *i*, a summation of the supply to demand ratios for the set of facilities falling within the threshold distance, *d*. Hence, prior to this step, each FCA metric contains disaggregated information regarding the spatial accessibility that is provided *by each facility* for that population unit; however, given that the ultimate goal of the FCA metrics is to capture an overall measure of spatial accessibility for population units, this information is summed to calculate the final *A*_*i*_ value for each (i.e., Eqs. 3, 5, and 9). Notably, the partial accessibility provided by each facility can be reconceptualized such that it describes the probability that people living in population unit *i* will visit facility *j* (*p*_*i,j*_), such that:

10 $$ {p}_{i,j}=\frac{A_{i,j}}{A_i} $$

In this formulation, normalizing each partial accessibility value (*A*_*i,j*_) by the total accessibility for the unit (*A*_*i*_) results in sets of *p*_*i,j*_ values that will always sum to one for each population unit and thus can be used to predict or estimate the proportion of each population unit that will use each facility. It is also important to note the connection between the *RI* values (Eq. 1) and the predicted probabilities from the partial spatial accessibility calculation in Eq. 10. The actual utilization patterns measured by *RI* values are directly comparable with the predicted utilization patterns based on spatial accessibility and represented as *p* values. This potentially highly valuable property of all FCA-based metrics has not been examined in previous research.

Potential spatial accessibility was calculated using four FCA metrics, the E2SFCA, M2SFCA, 3SFCA, and H3SFCA, using the number of hospital beds at each hospital as the measure of supply (*S*), the total population of each Zip Code as the potential demand (*P*), and the travel time from the PWC of each Zip Code to each hospital as the distance measure (*d*). The potential spatial accessibility values were converted to predicted probabilities of use per Eq. 10. We also calculated a simple distance-based measure of accessibility (DIST) using the selection weight formula from the 3SFCA (Eq. 6) and a simple Huff-based measure of accessibility (HUFF) using the selection weight formula from the H3SFCA (Eq. 7) for comparative purposes. The distance- and Huff-based accessibility values were also converted to predicted probabilities using Eq. 10.

### Distance decay functions

The FCA-, distance-, and Huff-based accessibility measures all require a threshold distance and a distance decay function. The threshold distance is the distance at which a facility is no longer considered accessible, which was set at 90 min to mirror the constraints placed on the hospital utilization data. The choice of the particular distance decay function and its parameter value(s) can have a large effect on the resulting accessibility scores, as this governs the conversion of measured distances (*d*) to weight values (*W*). As a sensitivity analysis, we calculated the metrics and corresponding predicted probabilities of utilization using four different decay functions, with each function having four unique parameter settings. The four functions were the Downward Log Logistic (DLL), Gaussian (GAUS), Exponential (EXP), and Logistic Cumulative Distance Function (LCDF), which have been used in similar work [7, 25, 33].

**Fig. 2:**
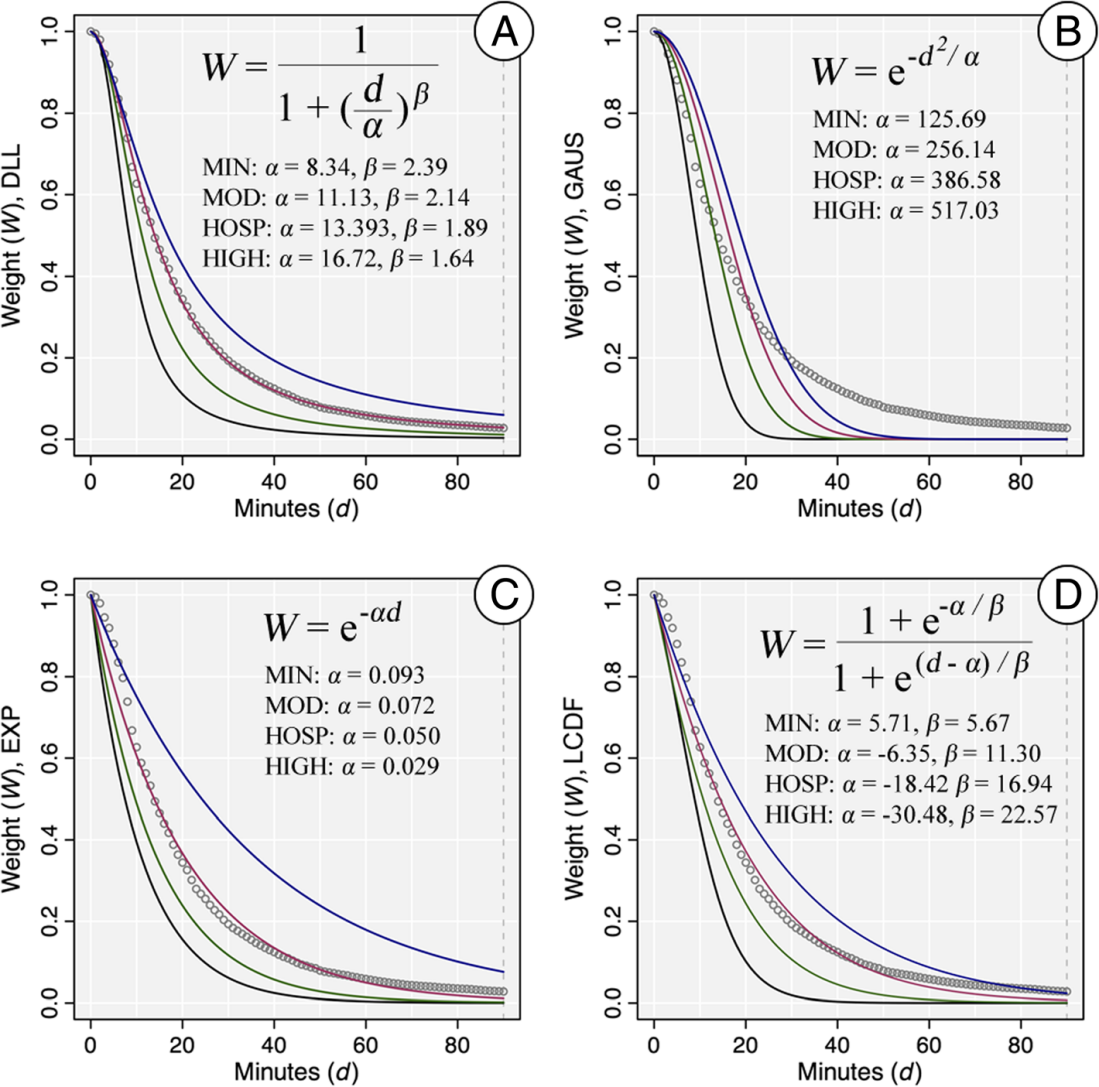
Weight values and formulas for the distance decay functions. The four function forms are **a**) Downward Log Logistic (DLL), **b**) Gaussian (GAUS), **c**) Exponential (EXP), and **d**) Logistic Cumulative Distance Function (LCDF). The four parameter settings for each function are MIN (black), MOD (green), HOSP (red), HIGH (blue). The observed distance decay from the hospital utilization data are plotted as grey circles for reference

The parameter settings we used for each function cover a broad range of potential distance decay relationships. The processes used to generate the parameter settings is summarized here and detailed in the Additional file 1. The first set of parameter values for the decay functions was based on an estimate of distance decay if each person in the state uses their nearest facility (MIN) and estimated by fitting the function to the data of the minimum distance to a facility. The next set of parameter values was based on the observed distance decay observed in the hospitalization data (HOSP) and estimated by fitting the function to the observed hospital utilization data. The third set of parameter values (MOD) was calculated by taking the mean of the parameter values of MIN and HOSP, which represents “moderate” distance decay. The fourth set of parameter values (HIGH) was calculated by adding the difference between the second and third set of values back to the third set of values. The HIGH set of values represents a “high miss” when estimating the observed distance decay behavior. The distance decay weights for the four functions, along with the formulas and four parameter settings, are presented in Fig. 2. As the figure shows, the four parameter settings cover a broad range of potential decay relationships for each function. While some of the function-parameter combinations fit the observed utilization data (e.g., DLL-HOSP), the combinations also include both functional forms and parameters settings that are very different from the observed distance decay. This range of both accurate and inaccurate combinations is important to evaluate, because the true distance decay relationship is generally not known when calculating potential spatial accessibility since utilization data are not available.

**Fig. 3:**
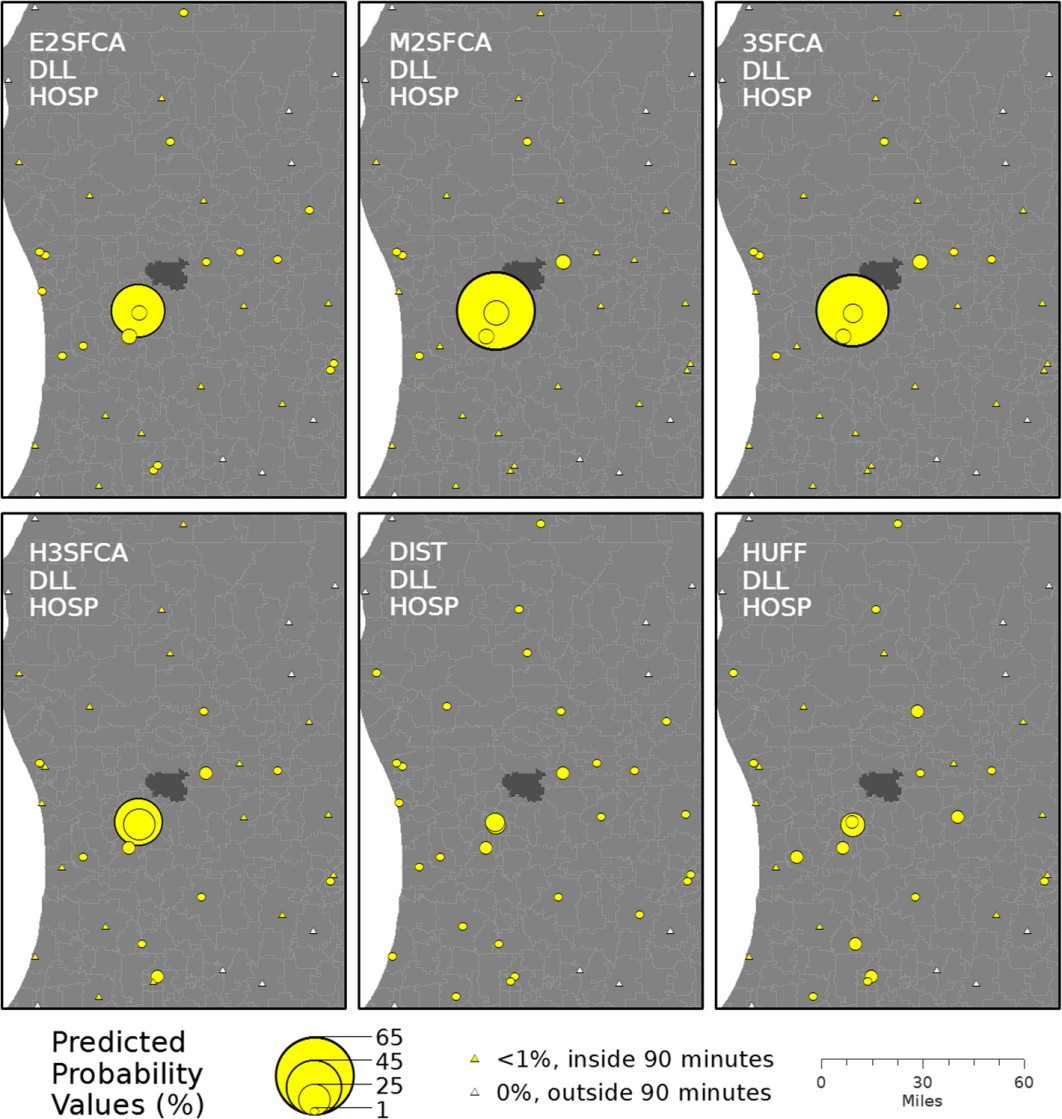
Predicted probabilities of hospital utilization for an example Zip Code. The example Zip Code is shaded dark. The probabilities were calculated using the six potential spatial accessibility metrics with a constant decay function (DLL) and set of parameters (HOSP)

To illustrate the differences in the predicted probabilities of use among the six metrics, they are mapped for a single example Zip Code in Fig. 3 (using the DLL function and HOSP parameter setting). Example figures showing the differences in predicted probabilities due to changes in the distance decay function and the decay function’s parameter settings can be found in Additional file 1.

### Comparison with observed utilization patterns

We assessed how well the six spatial accessibility metrics predicted observed hospital utilization patterns from the perspective of both the number of visits (counts) and the proportion of visits (*RI* values). This distinction was important because the overall accuracy of the predicted utilization patterns could be influenced by the different number of hospital visits that originate from each Zip Code; for example, a 5% error on 100 visits is 5 visits, whereas a 5% error on 1000 visits is 50 visits. By computing accuracy based on both absolute and normalized utilization patterns, we captured two related but distinctive properties of each spatial accessibility metric’s ability to predict utilization patterns. We also assessed the accuracy of assigning every patient to their nearest facility, as this approach is often used [15] and provides a useful benchmark to evaluate the predictive accuracy of the spatial accessibility metrics.

Because the potential spatial accessibility metrics only produced probabilities of utilizing each facility (Eq. 10), we generated predicted visit counts from each Zip Code to each facility by multiplying the total number of hospital visits for each Zip Code by the predicted probability values. As such, the total number of predicted hospital visits for each Zip Code was apportioned to facilities based on the probabilities of utilization gathered from each spatial accessibility metric.

To assess the accuracy of each spatial accessibility metric, we first calculated the percent of patient visits that were correctly predicted. For each Zip Code, the observed number of visits to each facility was subtracted from the predicted number of visits to calculate the prediction error. This resulted in a prediction error matrix containing both under and over predictions (negative and positive values) that summed to 0 because of the bound nature of visits. For example, if a single hospital visit was mistakenly assigned to Hospital A instead of Hospital B, this mistake would be recorded in the prediction error matrix as + 1 error at Hospital A and − 1 error at Hospital B. Hence, to calculate the percent correct based on counts, we first summed all of the positive prediction errors in the matrix, and then subtracted this sum from the total number of visits in the state. This calculation produced the number of visits that were correctly predicted, which was divided by the total number of visits to calculate the statewide percent of visits from each Zip Code to each Hospital that were correctly predicted. This calculation was performed for each spatial accessibility metric, decay function, parameter setting combination. An example of this calculation is provided in Additional file 1.

To calculate the percent correct based on the proportion of visits, we first used the above approach to calculate the percent correct for each Zip Code *separately*. Then, we calculated the mean percent correct over all Zip Codes. As such, this second measure of accuracy does not weigh by the differing number of visits originating from each Zip Code and therefore represents each spatial accessibility metric, decay function, parameter setting combination’s ability to predict normalized utilization patterns (*RI* values). Additional file 1 also contains an example of this calculation.

We performed a detailed analysis for a single metric, decay function, and parameter combination to better understand the spatial distribution of the errors of prediction and their potential causes. Notably, we wanted to understand whether the number of facilities in the local region or the distribution of utilization among facilities (e.g., see Fig. 1) affected the ability of the metrics to predict patterns of utilization for each Zip Code. We tabulated the number of facilities within 90 min of each Zip Code. We calculated the Shannon Evenness Index (*E*, [34, 35]) using the observed *RI* values of hospitals within 90 min of each Zip Code as a measure of the evenness of use across facilities. Potential *E* values range from 0 (all utilization at a single facility) to 1 (perfectly even distribution of utilization across multiple facilities).

## Results

The percent of visits correctly predicted based on counts for each spatial accessibility metric, decay function, and parameter combination are found in Table 1. The most accurate metric-function-parameter combinations were the 3SFCA-DLL-MOD (73.88% correct) and M2SFCA-DLL-MOD (73.84%), which were followed closely by the E2SFCA-LCDF-MIN (71.71%). The most accurate combinations for the three other metrics were each under 65%. The accuracy of the nearest facility approach was 38.9%, which nearly all metric-function-parameter combinations largely outperformed. The sensitivity test of the metrics to the decay functions and parameter settings also provided interesting findings. Notably, the range of the percent correct for the 3SFCA, M2SFCA, and H3SFCA were each less than 16% (less sensitive), while the E2SFCA, DIST, and HUFF metrics each had a range greater than 25% (more sensitive).

**Table 1:** Percent of hospital visits (based on counts) correctly predicted

METRIC, FUNCTION	MIN	MOD	HOSP	HIGH
E2SFCA, DLL	67.10	60.62	53.91	48.05
E2SFCA, GAUS	68.43	68.78	66.87	64.81
E2SFCA, EXP	67.59	62.69	54.67	43.88
E2SFCA, LCDF	**71.17**	65.11	56.72	50.41
M2SFCA, DLL	71.11	**73.84**	71.72	66.47
M2SFCA, GAUS	62.03	68.59	69.56	69.15
M2SFCA, EXP	72.46	72.81	69.91	58.59
M2SFCA, LCDF	67.82	72.49	70.47	66.31
3SFCA, DLL	71.62	**73.88**	71.58	66.52
3SFCA, GAUS	62.44	69.02	70.08	69.68
3SFCA, EXP	73.14	73.19	70.01	58.88
3SFCA, LCDF	68.45	73.02	70.63	66.54
H3SFCA, DLL	**61.83**	60.79	57.51	52.27
H3SFCA, GAUS	56.87	58.93	58.75	58.01
H3SFCA, EXP	61.15	60.12	56.23	46.29
H3SFCA, LCDF	59.95	60.36	57.12	52.65
DIST, DLL	57.78	51.08	44.43	38.90
DIST, GAUS	62.49	62.13	59.23	56.30
DIST, EXP	58.43	53.19	45.02	34.97
DIST, LCDF	**63.92**	55.79	47.05	40.97
HUFF, DLL	46.68	40.85	35.81	31.64
HUFF, GAUS	**53.98**	51.74	48.82	45.89
HUFF, EXP	47.49	42.61	36.24	28.69
HUFF, LCDF	53.31	44.91	37.77	33.23

Legend: The spatial accessibility metric and decay function are in the rows and the decay functions’ parameter settings are in the columns. The highest accuracy combination for each metric is in bold text

Table 2 contains the percent of visits correctly predicted based on proportions for each metric-function-parameter combination. Notably, the maximum values in Table 2 all are lower than the corresponding count-based results in Table 1, signaling that, in general, the accuracy of the spatial accessibility metrics was influenced by the raw count of visits. The M2SFCA, 3SFCA, and E2SFCA were again the most accurate in predicting utilization patterns. Interestingly though, the decay-parameter combinations with the most accurate results were not the same as the count-based results. The M2SFCA-DLL-HOSP (68.9% correct) and 3SFCA-EXP-HOSP (68.1%) were the most accurate in predicting normalized patterns of utilization, followed by the E2SFCA-EXP-MIN (66.14%). The H3SFCA, DIST, and HUFF metrics were lower in this measure of accuracy as well, having maximums of 57.78, 57.46, and 51.1% correct respectively. The nearest facility approach had an accuracy of 39.8% correct and, overall, the spatial accessibility metrics outperformed this approach. The sensitivity of the metrics to variations in the distance decay function and parameter was quite different using the normalized utilization patterns. The least sensitive metric was the H3SFCA with a range of only 7.68%, although all combinations were quite low in accuracy comparatively. The range of the other metrics was between 14.69 and 18.14%, which was similar to the count-based results.

**Table 2:** Percent of hospital visits (based on proportions) correctly predicted

METRIC, FUNCTION	MIN	MOD	HOSP	HIGH
E2SFCA, DLL	64.17	61.55	57.67	53.60
E2SFCA, GAUS	57.15	61.15	63.18	64.22
E2SFCA, EXP	**66.14**	64.53	59.85	51.45
E2SFCA, LCDF	63.62	65.57	61.43	56.95
M2SFCA, DLL	64.75	68.08	**68.90**	66.88
M2SFCA, GAUS	52.33	57.52	60.27	62.16
M2SFCA, EXP	64.31	67.03	68.53	63.28
M2SFCA, LCDF	57.82	65.45	68.23	67.66
3SFCA, DLL	64.60	67.05	67.65	65.99
3SFCA, GAUS	52.66	58.12	60.96	62.75
3SFCA, EXP	64.82	66.96	**68.10**	63.10
3SFCA, LCDF	58.57	65.70	67.93	67.35
H3SFCA, DLL	57.18	57.23	55.92	53.09
H3SFCA, GAUS	50.10	53.14	54.59	55.54
H3SFCA, EXP	57.28	**57.78**	56.56	50.55
H3SFCA, LCDF	54.04	57.43	57.11	54.95
DIST, DLL	53.65	49.99	45.76	41.75
DIST, GAUS	53.61	56.03	56.21	55.63
DIST, EXP	56.28	53.42	47.82	39.50
DIST, LCDF	**57.46**	55.18	49.57	44.90
HUFF, DLL	44.99	41.44	37.92	34.66
HUFF, GAUS	49.23	49.88	49.28	48.19
HUFF, EXP	48.15	44.88	39.78	32.97
HUFF, LCDF	**51.10**	46.80	41.34	37.34

Legend: The spatial accessibility metric and decay function are in the rows and the decay functions’ parameter settings are in the columns. The highest accuracy combination for each metric is in bold text

**Fig. 4:**
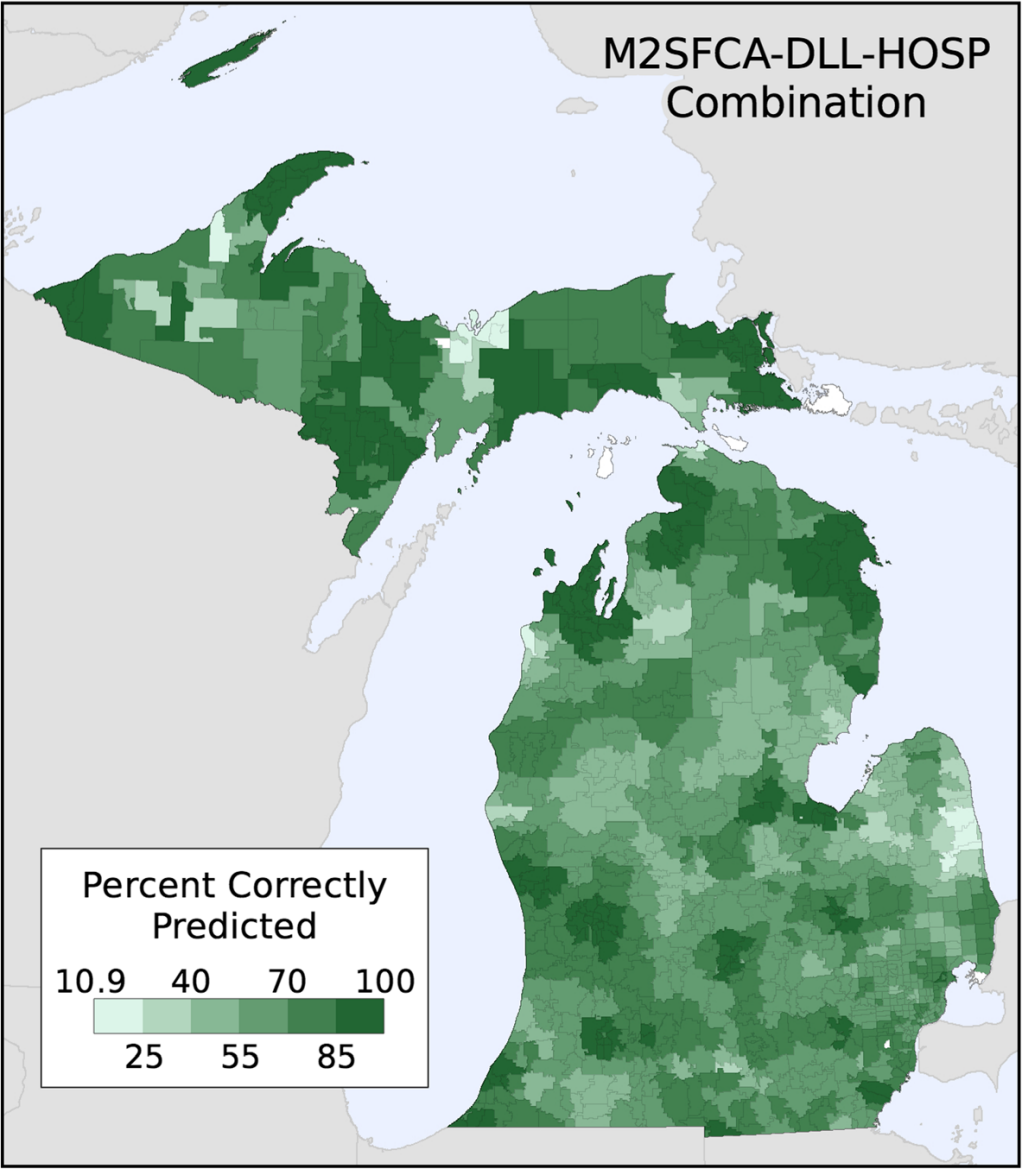
Percent of hospital visits correctly predicted for each Zip Code using the M2SFCA-DLL-HOSP combination

The percent of hospital visits correctly predicted by the M2SFCA-DLL-HOSP combination is mapped by Zip Code in Fig. 4. This combination was chosen because it was the best predictor of normalized utilization patterns and third highest predictor of the count-based utilization patterns. Using this metric, the minimum percent correctly predicted for any Zip Code was 10.9%, while the maximum was 99.9% (after removing a Zip Code with only a single hospital within 90 min because it was 100% correctly predicted). The map shows that there was high heterogeneity throughout the state, as this metric was very accurate in some regions and quite inaccurate in others.

**Fig. 5:**
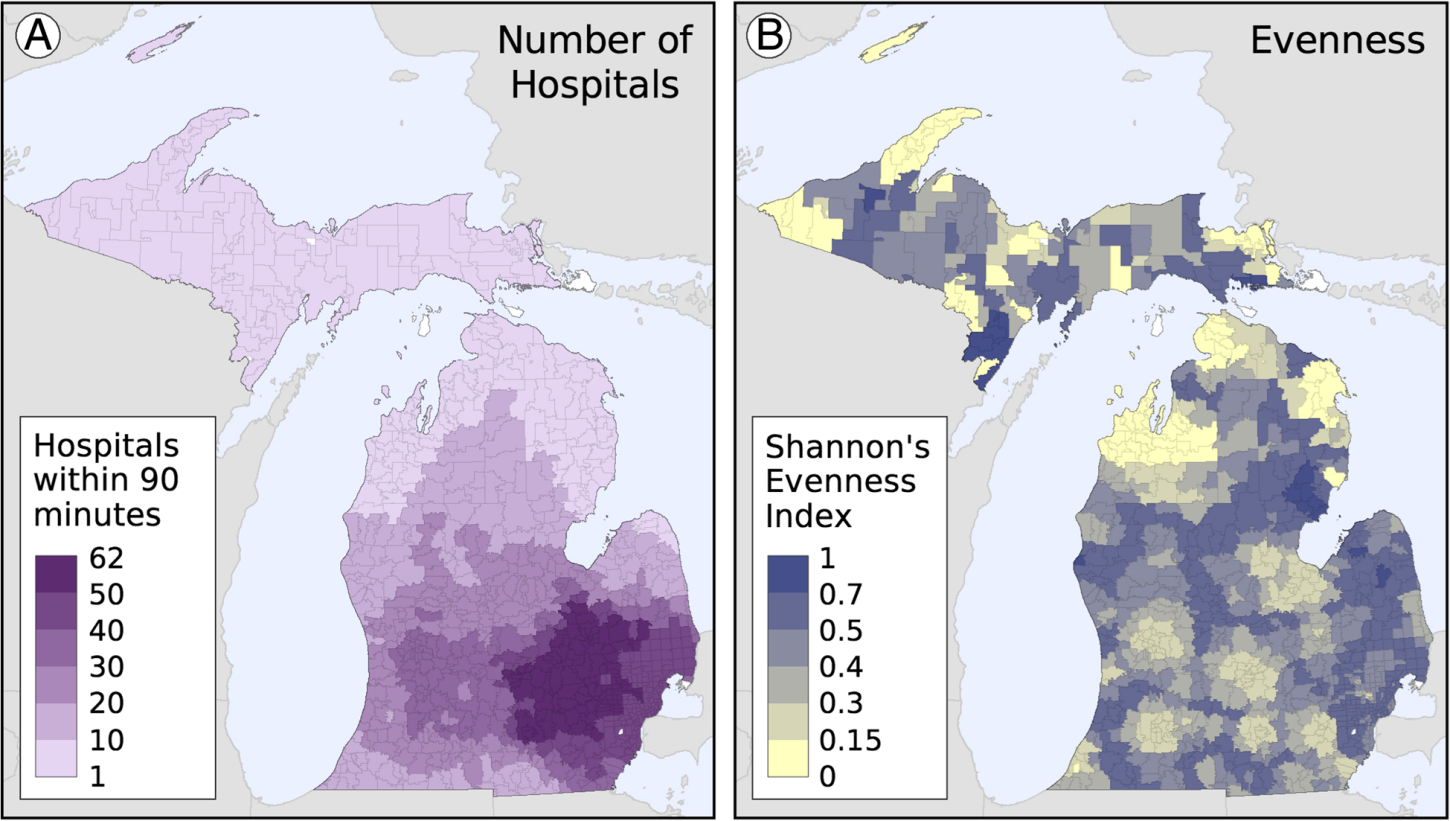
**a** The number of hospitals within 90 min and (**b**) Shannon’s Evenness Index values

Figure 5a shows the number of hospitals within 90 min of each Zip Code and Fig. 5b shows the Shannon’s Evenness Index values based on observed utilization patterns. The concentration of hospitals in the southeast portion of Michigan is apparent, which is where much of the state’s population resides. The places with the highest number of hospitals within 90 min are located in the region between metro Detroit, Lansing, Ann Arbor, and Saginaw. The most obvious pattern in the evenness map are the low values (indicating utilization largely occurs at a single or very few facilities) found in and around population centers in the southern part of the state, which have a small number of large hospitals and the low values in and around the larger, regional hospitals found throughout the northern part of the state. High values of evenness are found in many of the regions located “in between” population centers, indicating utilization is distributed among multiple hospitals.

**Fig. 6:**
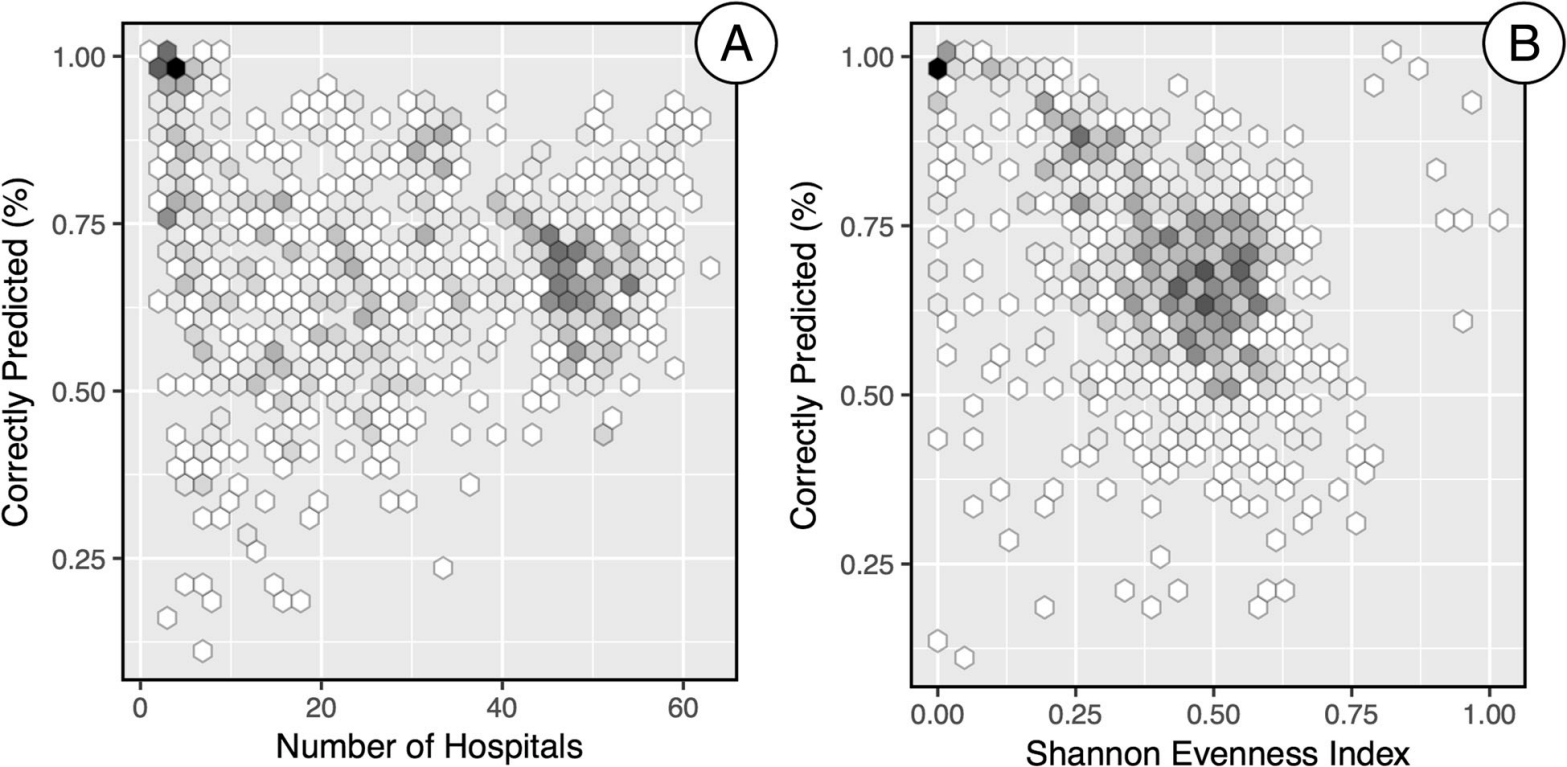
Percent of hospital visits correctly predicted for each Zip Code. Predictions were made using the M2SFCA-DLL-HOSP combination plotted against (**a**) the number of hospitals within 90 min of each Zip Code and (**b**) the Shannon’s Evenness Index values. The number of points in each hexagon ranges from 1 (white) to 18 (black)

The percent of hospital visits correctly predicted using the M2SFCA-DLL-HOSP combination is plotted against the number of hospitals within 90 min of each Zip Code in Fig. 6a and against the Shannon Evenness Index values in Fig. 6b. The plots do not provide strong evidence of a systematic effect on the M2SFCA’s ability to predict patterns of utilization based on the potential number of hospitals or the evenness of utilization across hospitals. Interestingly, the greatest range in predictive accuracy is found at the lower end of the distribution of both variables. This is a somewhat counterintuitive finding in that fewer hospitals nearby and more concentrated utilization across hospitals could be considered a less complex scenario (than evenly distributed hospital visits across numerous hospitals) to attempt to predict.

## Discussion

The results of the analysis demonstrate that the disaggregated information contained within FCA metrics serves as a viable model for predicting geographic utilization patterns when this information is unavailable, as is often the case. Numerous metric-function-parameter combinations were able to correctly predict over 70% of all inpatient hospital visits occurring within 90 min for an entire state over a single calendar year, which is quite remarkable given that the case study included predicting more than a million hospital visits across more than 25,000 potential origin destination pairs. All of the FCA metrics provided substantial gains in predictive accuracy over the simple nearest facility approach. Specifically, the E2SFCA, 3SFCA, and M2SFCA produced the most accurate predictions using count-based and normalized utilization patterns.

Our results suggest that FCA metrics have potential for use in estimating where people go to receive health care in the absence of detailed utilization data and as an alternative to the assumption that people use the nearest facility. This information can be useful in a number of circumstances. First, it can be directly applied for planning purposes; specifically, if some regions are expected to need a higher volume of services in the future, estimated utilization patterns could be used to predict which facilities may require more resources to meet that future demand.

Another potential use is to use utilization pattern estimates in understanding health outcomes as they relate to where people are receiving health services. A common issue faced in health services research is how to account for variations in facility quality or other aspects of service provision when examining population-level utilization and health outcomes. For this purpose, the estimated utilization patterns can be integrated within an analysis to assign facility-level information to populations based on the estimated proportion of people using each facility.

Another circumstance in which our findings may be useful is when a researcher has utilization data at the facility-level, but does not have information about the residential location of the people who visited each facility. Whereas our work focused on predicting the *destination* aspect of utilization patterns, the approach could be reconfigured to predict patient *origins*. While this would require further analysis and evaluation of this modified approach, it has the potential to provide valuable estimates of where patients originated in the absence of this information. Specifically, it might be used by facilities to better understand the underlying population from which their patient base is drawn.

The results of the sensitivity analysis showed that the accuracy of the predictions for all metrics was affected by the choice of the particular distance decay function and its parameters. A known limitation of spatial accessibility metrics that use distance decay in their formulation is that the output will vary given changes to the decay function or parameter settings [24, 36, 37]. Further, there is often little guidance from prior research to justify using one function or parameter setting over another. It is difficult to disentangle the reasons why some of the metrics were more affected by the variations in distance decay than others because the decay function, its parameters, and the metric’s formulation all are working simultaneously to characterize spatial accessibility. Because most researchers will not have access to observed data that can be used to evaluate the accuracy of utilization predictions made using spatial accessibility metrics, we suggest caution in using FCA metrics for predicting utilization patterns without considering this limitation. However, an important takeaway from the sensitivity analysis is that many of the metrics’ predictions were more accurate when the distance decay function parameter(s) resulted in a stronger decay effect. The MIN parameter set generally produced more accurate results than the HIGH parameter set (high miss) in both the count-based and normalized predictions for a number of metrics. This finding is important because the MIN parameter set does not require any utilization data to be estimated; it only requires population counts (and locations), facility locations, and the distance separating people and facilities, which are the same inputs needed to calculate an FCA metric and can be readily obtained for many study areas. Another noteworthy result is that the HOSP parameter set, which was derived directly from the utilization data, did not always provide the most accurate results. This suggests that knowing the globally averaged distance decay parameter set may not capture local variations in this relationship.

While the state-level accuracy of the M2SFCA-DLL-HOSP combination was quite high, the detailed analysis (e.g., the map in Fig. 4) showed that there was a large variation in the predictive accuracy of local patterns of utilization. We evaluated whether this was a function of the number of hospitals near each Zip Code or the evenness of utilization across multiple hospitals and did not find any strong evidence of a systematic relationship (Fig. 6). The variation in predictive accuracy is likely due to non-spatial local factors (both population- and facility-level) that influence where people seek health care, which are generally not considered in spatial accessibility models such as FCA metrics that largely ignore differences among populations (e.g., treated equally in the potential demand step), facilities (e.g., treated equally in the supply step), and distance decay behavior (use of a single distance decay parameter for the entire model). While this simplification offers computational advantages in a model of “potential” spatial accessibility, it may not be entirely appropriate for predicting local patterns of realized utilization. Further research is needed to better understand why spatial accessibility did or did not provide accurate predictions in some locations. Another future research direction would be too leverage this information (local predictive accuracy) in an effort to better understand and potentially improve health care delivery in a study area. A possible example includes identifying regions where residents are traveling further than expected to access services and examining whether this may be due to non-spatial barriers to accessing care, such as whether the nearby facilities offers the services required to meet the needs of the population.

### Limitations

While this research did shed light on the overall potential of using FCA metrics to predict utilization patterns, it does have limitations. First, the case study only considered acute care hospitalizations in a single state in the US. The predictive power of FCA metrics, along with other empirical findings of this study, are surely rooted within the context of Michigan’s health care system and the socioeconomic, cultural, and geographic context of the communities this system serves. Whether FCA metrics can predict utilization patterns for other health care services or in other US states or in other countries with highly different health care systems remains unknown but does present an avenue for further exploration.

A second limitation of this work was the omission of other factors known to influence where and how far patients travel to receive hospital care in the US, including their ability to travel (mobility), their health insurance status, different travel modes available (e.g., public transit and personal vehicles), the services provided at each hospital, the perceived or measured quality of the hospitals, doctor admitting privileges, and others. These omissions were part of the research design for this work, as our aim was to evaluate the utility of a relatively naïve, oft-encountered, and easy to implement model (FCA metrics) to predict use. As such, understanding how these other factors influence patient utilization patterns and whether they interact with potential spatial accessibility is an important opportunity for future research. Specifically, determining whether these factors played a role in the geographic variation in the predictive ability of the FCA metrics may potentially uncover how other aspects of potential access to care affect where people utilize facilities.

A third limitation of the analysis was the use of the number of bed licenses as a proxy for supply in the spatial accessibility metrics. While this measure is often used, it does not always capture a facility’s actual supply or ability to provide care (e.g., if beds are not in use due to understaffing). In our study, we attempted to use as little auxiliary information as possible to mimic the conditions researchers would generally face when attempting to estimate utilization patterns from publicly-available facility data. Another data-related limitation is that three hospitals were excluded from the analysis, as was one Zip Code, due to lack of data; however, the missing data from these facilities and Zip Code represent a miniscule fraction of the statewide hospitalization and we do not believe this had any effect on our results.

Another limitation is that our analysis demonstrated that the predictive accuracy of the FCA metrics was sensitive to the particular metric, distance decay function, and the decay function parameters. Thus, we are not able to provide a definitive answer to which combination should be used in other circumstances, especially when researchers do not have the observed data to evaluate the accuracy of the predictions. While this does restrict the overall usefulness of this approach, we are encouraged that all four FCA metrics were much more accurate than assuming people visited their nearest facility, an approach that is oft-employed in these types of scenarios. Furthermore, the MIN distance decay parameter set performed quite well across metrics and decay functions. This is important because this parameter set is based on the distance from populations to facilities and does not require any additional data beyond what is necessary to calculate the FCA metrics. Thus, this parameter set does not require utilization data and can be calculated in any scenario when the location of people and facilities are known.

## Conclusions

The goal of this research was to provide a starting point to begin rectifying Higgs’ [1] assertion that the geographic aspects of health care research have largely favored potential access to care, rather than actual utilization of care. Our work focused on building a bridge between measures of potential spatial accessibility and observed geographic utilization patterns, showing that FCA metrics are able to provide reasonable predictions of these patterns. Using acute care hospital visits in Michigan, a number of the metrics evaluated were able to predict the correct destination hospital for more than 70% of the state’s roughly one million hospitalizations in 2014. This research also began exploring the local nature of the predictive ability, finding that the number of hospitals within 90 min of each region and the evenness of the region’s hospital visits across facilities did not appear to systematically affect the predictions. While our work did provide interesting results regarding the relationship between access to and utilization of health care, it also generated a number of important questions that deserve further exploration. In particular, additional exploration for different health care services and in different region would help to determine the range of predictive capacity for FCA metrics and the generalizability of findings from this empirical study.

## Additional file

Additional file 1: Distance decay parameter generation methods and examples. (DOCX 410 kb)

## Abbreviations

2SFCA: Two step floating catchment area
3SFCA: Three step floating catchment area
DIST: Simple distance-based measure of accessibility
DLL: Downward log logistic distance decay function
E2SFCA: Enhanced two step floating catchment area
EXP: Exponential distance decay function
FCA: Floating catchment area
GAUS: Gaussian distance decay function
H3SFCA: Huff-modified three step floating catchment area
HIGH: Parameter values for the decay functions representing a “high miss” when estimating the observed distance decay behavior
HOSP: Parameter values for the decay functions based on the empirical distance decay observed in the hospital utilization data
HUFF: Huff-based model of accessibility
LCDF: Logistic cumulative distance decay function
M2SFCA: Modified two step floating catchment area
MDHHS: Michigan Department of Health and Human Services
MIDB: Michigan inpatient database
MIN: Parameter values for the decay functions based on an estimate of distance decay if each person in the state used the nearest facility
MOD: Parameter values for the decay functions representing a “moderate” distance decay
OD: Origin-destination
PWC: Population weighted centroid
RI: Relevance index
