# Supplementary material for: Using floating catchment area (FCA) metrics to predict health care utilization patterns
Source: BMC Health Serv Res. 2019 Mar 4;19:144. doi: 10.1186/s12913-019-3969-5 (PMC6399985; doi:10.1186/s12913-019-3969-5)
Supplement: Supplementary file 1 — Distance decay parameter generation methods and examples. (DOCX 410 kb) [file 12913_2019_3969_MOESM1_ESM.docx]

**Using Floating Catchment Area (FCA) metrics to predict health care utilization patterns**

Paul L. Delamater, Ashton M. Shortridge, Rachel C Kilcoyne

**Additional File 1**

**A.1 Distance decay parameter generation methods**

We calculated the spatial accessibility metrics and corresponding predicted probabilities of utilization at each hospital using four different decay functions. For spatial accessibility metrics, the distance decay function converts the distance measurement (*d*), in our case measured as minutes of travel time, to a weight value (*W*) to be used in the spatial accessibility metric calculation. The four functions were the Downward Log Logistic (DLL),

$W= \frac{1}{1+ \left( \frac{d}{\alpha} \right)^{\beta}}$ ,

Gaussian (GAUS),

$W= e^{{{-d}^{2}}/\alpha}$ ,

Exponential (EXP),

$W= e^{-\alpha d}$ ,

and Logistic Cumulative Distance Function (LCDF),

$W= \frac{1+ e^{{-\alpha}/\beta}}{1+ e^{\left( d-\alpha\right)/\beta}}$ .

For each function, we used four unique parameter settings. The use of multiple distance decay functions and parameter settings were intended to cover a broad range of potential decay relationships, as observed data that can be used to estimate the true distance decay relationship is not often available when calculating potential spatial accessibility metrics.

The first set of parameter values for the decay functions was based on an estimate of distance decay if each person in the state used the nearest hospital (MIN). To estimate this function, we first calculated the cumulative probability of a person visiting a hospital that was within *d* minutes or more, using one-minute bins. For example, using the nearest hospital, 100% of people would travel 0 minutes or more to reach the nearest hospital (probability = 1), 98.83% of people would travel 1 minute or more (probability = 0.9883), and so on. We then used the non-linear least squares estimator available in R to estimate the parameter settings for each function, using the cumulative probability values as the Y variable and the distance as the X variable. The cumulative probabilities essentially function as an estimate of the *W* value in a distance decay function. The observed data, as well as the best fit lines for each function, are provided in Figure A.1.


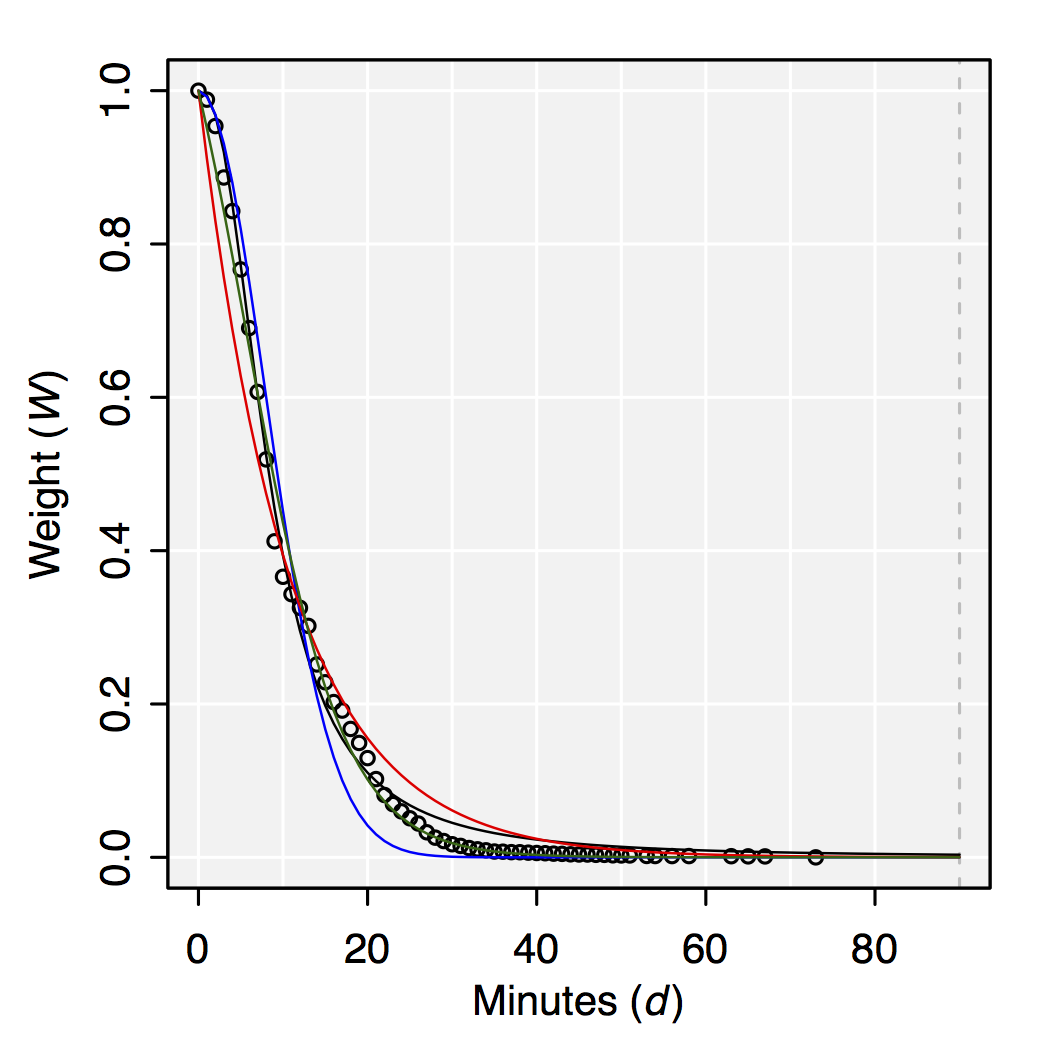


Figure A.1. Observed cumulative probability values based on distance to the nearest hospital and estimated distance decay functions, Downward Log Logistic (DLL, black), Gaussian (GAUS, blue), Exponential (EXP, red), and Logistic Cumulative Distance Function (LCDF, green).

The next set of parameter values was based on the observed distance decay relationship in the hospital utilization data (HOSP). Again, we calculated the cumulative probability of a person visiting a hospital that was within *d* minutes or more, using one-minute bins. In this case, we used the actual distance traveled information, gleaned from the utilization data. The observed data, as well as the best fit lines for each function, are provided in Figure A.2.


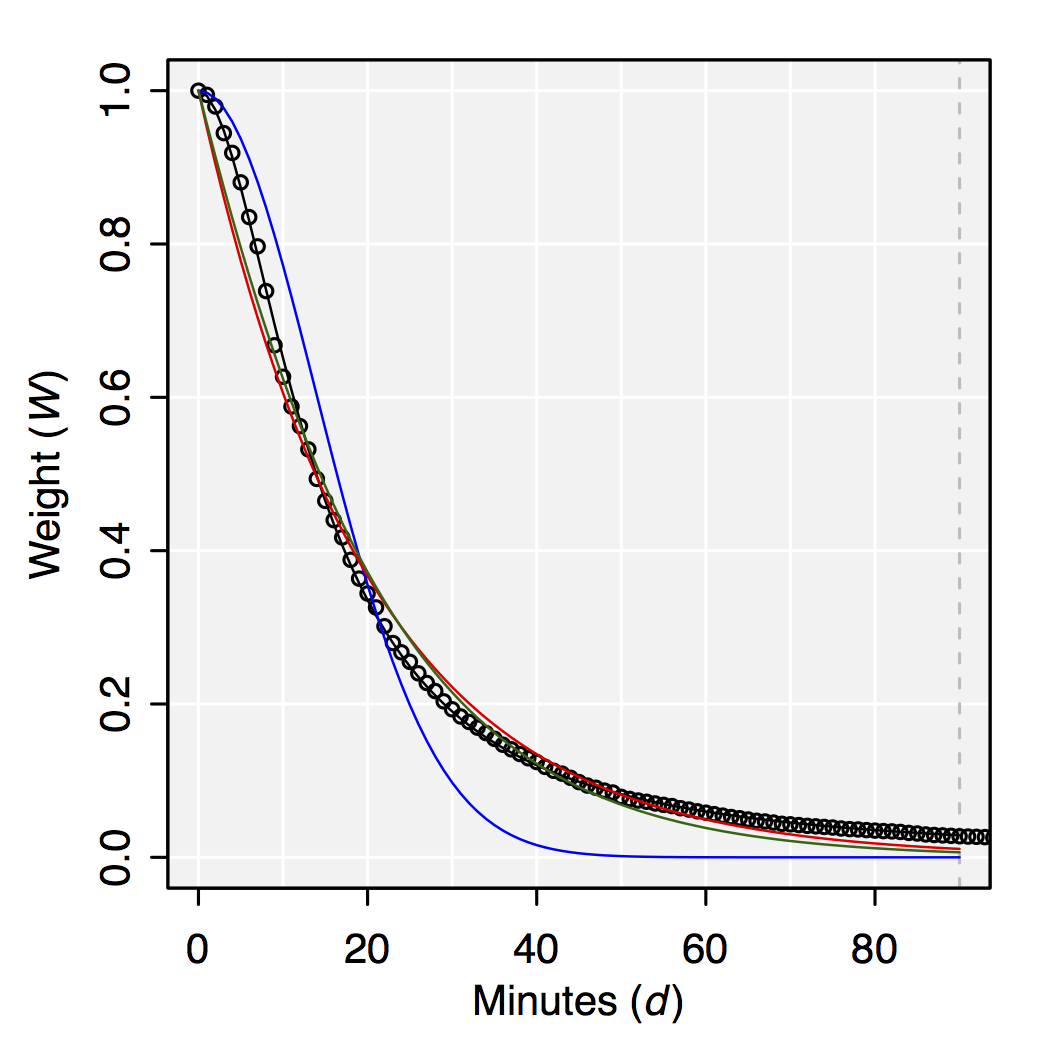


Figure A.2. Observed cumulative probability values based on observed distance traveled and estimated distance decay functions, Downward Log Logistic (DLL, black), Gaussian (GAUS, blue), Exponential (EXP, red), and Logistic Cumulative Distance Function (LCDF, green).

The third set of parameter values (MOD) was intended to capture a distance decay relationship that fell in between the minimum distance (MIN) and actual distance (HOSP). It was calculated by taking the mean of the parameter values of MIN and HOSP for each function and represents “moderate” distance decay. The fourth set of parameter values (HIGH) was intended to represent a “high miss” when estimating the distance decay relationship. This set of parameter values was calculated by adding the difference between the second (MOD) and third (HOSP) set of values back to the third set of values.

The four sets of parameter values, as well as the regression fit (*R^2^*) for MIN and HOSP, are provided in Table A.1 for each decay function.

| **Function, parameter set** | **𝛼** | **𝛽** | ***R^2^*** |
| --- | --- | --- | --- |
| DLL, MIN | 8.342 | 2.387 | 0.995 |
| DLL, MOD | 11.135 | 2.137 | NA |
| DLL, HOSP | 13.927 | 1.887 | 0.999 |
| DLL, HIGH | 16.719 | 1.638 | NA |
| GAUS, MIN | 125.688 | NA | 0.978 |
| GAUS, MOD | 256.136 | NA | NA |
| GAUS, HOSP | 386.584 | NA | 0.897 |
| GAUS, HIGH | 517.031 | NA | NA |
| EXP, MIN | 0.093 | NA | 0.972 |
| EXP, MOD | 0.072 | NA | NA |
| EXP, HOSP | 0.050 | NA | 0.981 |
| EXP, HIGH | 0.029 | NA | NA |
| LCDF, MIN | 5.710 | 5.667 | 0.994 |
| LCDF, MOD | -6.353 | 11.302 | NA |
| LCDF, HOSP | -18.416 | 16.938 | 0.983 |
| LCDF, HIGH | -30.479 | 22.573 | NA |

Table A.1. Summary results for distance decay functions, Downward Log Logistic (DLL), Gaussian (GAUS), Exponential (EXP), and Logistic Cumulative Distance Function (LCDF) and parameter settings.

**A.2 Examples of the effect of distance decay function and parameter settings on predicted probabilities**

To demonstrate the differences in predicted probabilities due to variations in the distance decay function, predicted probabilities of utilization from the M2SFCA are mapped for a single Zip Code in Figure A.3 (using the HOSP parameter setting for each). To highlight the variations in probabilities that result from changes to the distance decay function’s parameter settings, the predicted probabilities of utilization from the M2SFCA are mapped in Figure A.4 for a single Zip Code using a single function (DLL) with the four different parameter settings.


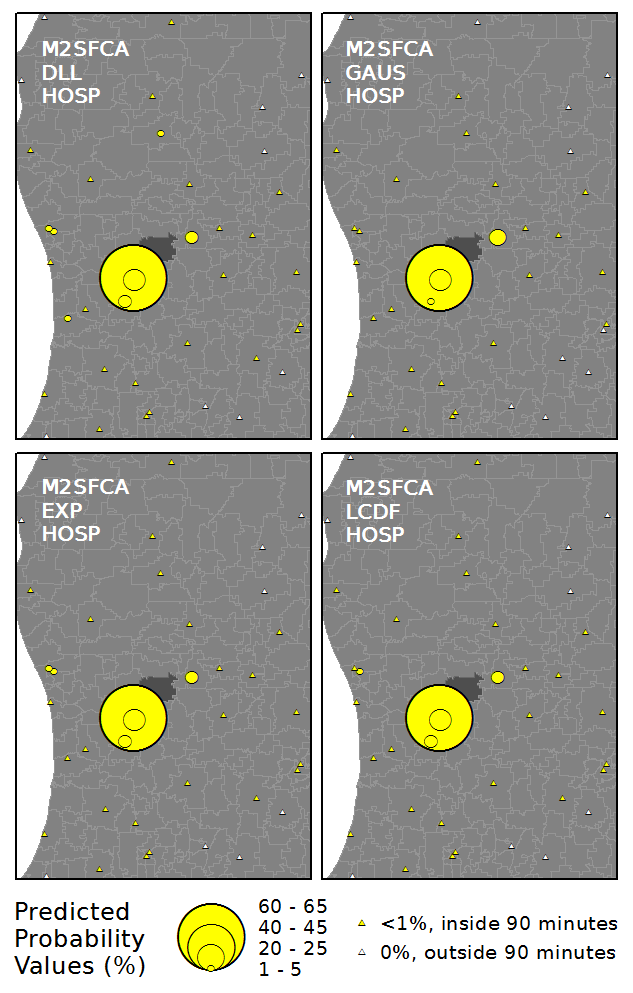


Figure A.3. Predicted probabilities of hospital utilization for an example Zip Code (dark), using the M2SFCA metric with four different distance decay functions and a constant set of parameters (HOSP).


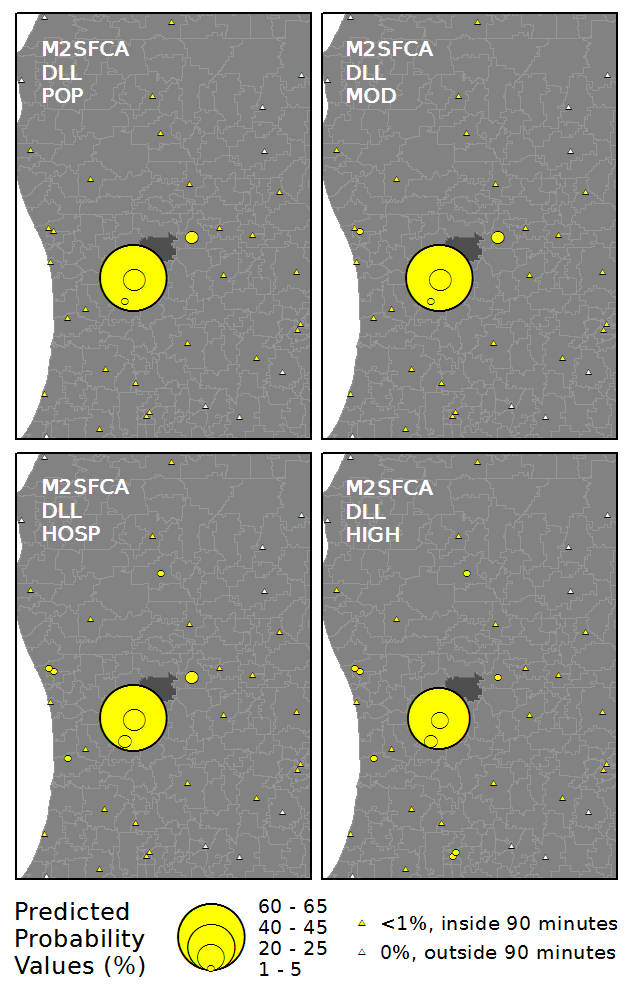


Figure A.4. Predicted probabilities of hospital utilization for an example Zip Code (dark), using the M2SFCA metric with a single decay function (DLL) and four sets of parameters.

**A.3 Example of percent of patient visits correctly predicted (for counts and proportions)**

The accuracy assessment we used was based on the percent of hospital visits that were correctly predicted. For each Zip Code, the observed number of visits to each facility was subtracted from the predicted number of visits. The output of this calculation is a set of prediction errors containing both under and over predictions (negative and positive values) that summed to 0 because of the bound nature of the visits. For illustrative purposes, an example calculation for a hypothetical system of 5 Zip Codes and 3 hospitals is provided below in Tables A.2 (Observed Hospital Visits), A.3 (Predicted Visits), and A.4 (Prediction Errors).

| **Zip Code** | **H1** | **H2** | **H3** | ***SUM*** |
| --- | --- | --- | --- | --- |
| Z1 | 25 | 25 | 50 | *100* |
| Z2 | 15 | 45 | 20 | *80* |
| Z3 | 40 | 50 | 60 | *150* |
| Z4 | 10 | 5 | 5 | *20* |
| Z5 | 80 | 10 | 30 | *120* |

Table A.2. Observed hospital visits Origin-Destination matrix for the example system. The table entries contain the number of hospital visits. Sum is the sum of visits for each Zip Code.

| **Zip Code** | **H1** | **H2** | **H3** | ***SUM*** |
| --- | --- | --- | --- | --- |
| Z1 | 30 | 25 | 45 | *100* |
| Z2 | 10 | 50 | 20 | *80* |
| Z3 | 50 | 55 | 45 | *150* |
| Z4 | 5 | 0 | 15 | *20* |
| Z5 | 85 | 5 | 30 | *120* |

Table A.3. Predicted hospital visits Origin-Destination matrix for the example system. The table entries contain the number of hospital visits. Sum is the sum of visits for each Zip Code.

| **Zip Code** | **H1** | **H2** | **H3** | ***SUM*** |
| --- | --- | --- | --- | --- |
| Z1 | 5 | 0 | -5 | 0 |
| Z2 | -5 | 5 | 0 | 0 |
| Z3 | 10 | 5 | -15 | 0 |
| Z4 | -5 | -5 | 10 | 0 |
| Z5 | 5 | -5 | 0 | 0 |

Table A.4. Prediction errors for the example system. The table entries contain the number of hospital visits incorrectly predicted. Sum is the sum of visits for each Zip Code and equals 0 due to the bound nature of hospital visits.

As a result, any error in prediction shows up as two “errors” in the table (+1 to the erroneous hospital and -1 to the hospital actually visited). To calculate the percent correct for the entire state, we first summed all of the positive prediction errors in the matrix (in Table A.4), and then subtracted this sum from the total number of visits in the state. This calculation produced the number of visits that were correctly predicted, which was divided by the total number of visits to calculate the statewide percent of visits from each Zip Code to each Hospital that were correctly predicted. In the example above, this calculation would be 40 (sum of positive errors in Table A.4) subtracted from 470 (sum of all visits in Table A.2), which results in 430 visits correctly predicted. Then, 430 divided by 470 equals 91.49% of all visits correctly predicted.

To calculate the percent correct based on the proportion of visits (i.e., the Relevance Index values), we first used the above approach to calculate the percent correct for each Zip Code using the approach described above. For demonstration purposes, the actual and predicted Relevance Index (RI) values for the example system can be found in Tables A.5 and A.6, respectively. Table A.7 contains the values used in the calculation.

| **Zip Code** | **H1** | **H2** | **H3** |
| --- | --- | --- | --- |
| Z1 | 0.25 | 0.25 | 0.50 |
| Z2 | 0.19 | 0.56 | 0.25 |
| Z3 | 0.27 | 0.33 | 0.40 |
| Z4 | 0.50 | 0.25 | 0.25 |
| Z5 | 0.67 | 0.08 | 0.25 |

Table A.5. RI values for each Zip Code and hospital pair based on the observed hospital visits for the example system.

| **Zip Code** | **H1** | **H2** | **H3** |
| --- | --- | --- | --- |
| Z1 | 0.30 | 0.25 | 0.45 |
| Z2 | 0.13 | 0.63 | 0.25 |
| Z3 | 0.33 | 0.37 | 0.30 |
| Z4 | 0.25 | 0.00 | 0.75 |
| Z5 | 0.71 | 0.04 | 0.25 |

Table A.6. RI values for each Zip Code and hospital pair based on the predicted hospital visits for the example system.

| **Zip Code** | **SUM** | **CORR** | **PCT** |
| --- | --- | --- | --- |
| Z1 | 100 | 95 | 95.00 |
| Z2 | 80 | 75 | 93.75 |
| Z3 | 150 | 135 | 90.00 |
| Z4 | 20 | 15 | 75.00 |
| Z5 | 120 | 115 | 95.83 |

Table A.7. Values used in the calculation of the percent correctly predicted based on normalized utilization patterns (RI values).

To get the value for the entire state, we calculated the mean percent correctly classified for all Zip Codes. Because this second measure does not consider the differing number of visits originating from each Zip Code, it represents each spatial accessibility metric, decay function, parameter setting combination’s ability to predict normalized utilization patterns (*RI* values). In the example system, this would result in 89.92% of the proportion of visits correctly predicted. In the example, this value is lower than the count-based result due to the influence of Z4, which has a low value (75% correct), which highly affects the proportion-based results more because it is weighed equally to the other Zip Codes even though it only had 20 hospital visits in total.
